# Predictors of prenatal care satisfaction among pregnant women in American Samoa

**DOI:** 10.1186/s12884-017-1563-6

**Published:** 2017-11-16

**Authors:** Oluwaseyi Adeyinka, Anne Marie Jukic, Stephen T. McGarvey, Bethel T. Muasau-Howard, Mata’uitafa Faiai, Nicola L. Hawley

**Affiliations:** 1 Department of Chronic Disease Epidemiology, School of Public Health, Yale University, 60 College Street, New Haven, CT 06520-8034 USA; 2 International Health Institute, Department of Epidemiology, School of Public Health, Brown University, Providence, USA; 3 Lyndon B Johnson Tropical Medical Center, Pago Pago, American Samoa; 4 Division of Natural Sciences and Mathematics, Chaminade University, Honolulu, USA

**Keywords:** Prenatal care, Satisfaction, American Samoa, Physician interactions, Clinic accessibility

## Abstract

**Background:**

Pregnant women in American Samoa have a high risk of complications due to overweight and obesity. Prenatal care can mitigate the risk, however many women do not seek adequate care during pregnancy. Low utilization of prenatal care may stem from low levels of satisfaction with services offered. Our objective was to identify predictors of prenatal care satisfaction in American Samoa.

**Methods:**

A structured survey was distributed to 165 pregnant women receiving prenatal care at the Lyndon B Johnson Tropical Medical Center, Pago Pago. Women self-reported demographic characteristics, pregnancy history, and satisfaction with prenatal care. Domains of satisfaction were extracted using principal components analysis. Scores were summed across each domain. Linear regression was used to examine associations between maternal characteristics and the summed scores within individual domains and for overall satisfaction.

**Result:**

Three domains of satisfaction were identified: satisfaction with clinic services, clinic accessibility, and physician interactions. Waiting ≥ 2 h to see the doctor negatively impacted satisfaction with clinic services, clinic accessibility, and overall satisfaction. Living > 20 min from the clinic was associated with lower clinic accessibility, physician interactions, and overall satisfaction. Women who were employed/on maternity leave had lower scores for physician interactions compared with unemployed women/students. Women who did not attend all their appointments had lower overall satisfaction scores.

**Conclusions:**

Satisfaction with clinic services, clinic accessibility and physician interactions are important contributors to prenatal care satisfaction. To improve patient satisfaction prenatal care clinics should focus on making it easier for women to reach clinics, improving waiting times, and increasing time with providers.

**Electronic supplementary material:**

The online version of this article (10.1186/s12884-017-1563-6) contains supplementary material, which is available to authorized users.

## Background

Obesity, either before or during pregnancy, is an established risk factor for a number of maternal and fetal health complications [1–3]. Obesity in pregnant women is associated with increased incidence of preeclampsia, gestational diabetes, fetal macrosomia, and stillbirth [4]. In spite of this, the proportion of United States women of reproductive age who are overweight or obese at the time of conception continues to climb, mirroring trends among the general population; approximately 60% of women of reproductive age are overweight or obese [5–7]. Comparatively, as a result of rapid demographic and nutritional transition, almost 90% of American Samoan women of childbearing age are overweight or obese [8].

American Samoa is an unincorporated island territory of the United States located ~2400 miles southwest of Hawaii. The population receives benefits from its affiliation with the US such as the Special Supplemental Nutrition Program for Women, Infants, and Children (WIC) and Medicaid access, but the island remains a medically underserved and health care professional shortage area [9, 10]. Prenatal care is considered essential for a healthy pregnancy [11] but is particularly important in the American Samoan setting to ensure that women enter prenatal care early and receive quality care so that some of the risk associated with overweight/obesity during pregnancy can be mitigated. Gestational diabetes and hypertensive disorders of pregnancy, related to overweight/obesity, are both prevalent issues among those of Samoan ethnicity [12, 13] and require early detection and continued management.

Hawley et al. [14] used clinical record data from American Samoa to describe prenatal care utilization among women receiving care at the main hospital and one of the community health centers in American Samoa between 2001 and 2008. Using Kotelchuck’s criteria for adequacy of prenatal care [15] they found that few women (less than 25%) received adequate care during pregnancy, based on the timing of entry into care and the number of appointments attended.

Several factors determine pregnant women’s utilization of prenatal care services during pregnancy. Known barriers to prenatal care in American Samoa mirror those that have been established globally, such as financial barriers (unemployment, lack of transportation), lack of child care for other children, and late pregnancy recognition [14]. However, one factor that has not yet been explored as a barrier to adequate prenatal care utilization in American Samoa is satisfaction with prenatal care services. In other settings it has been shown that a woman who is dissatisfied with her prenatal care, specifically with the patient-provider interaction, is less likely to follow the prenatal care regimen; she is also less likely to utilize prenatal care in future pregnancies [16–19]. In addition, across all types of medical care, Chemir, Alemseged, and Workneh [20] explain that, “a satisfied patient will recommend [a] center’s services, expressing their satisfaction to four or five people, while a dissatisfied patient, on the other hand, will complain to twenty or more”, suggesting that dissatisfaction with prenatal care may impact both individual behavior and the behavior of a woman’s peers. Prior research exploring prenatal care satisfaction in other settings has found that socio-demographic characteristics such as race/ethnicity, occupation, educational attainment, and religion are significantly associated with satisfaction with care [20–24]. These factors have not been investigated in American Samoa.

This paper describes factors associated with satisfaction with prenatal care in American Samoa and identifies specific patient groups that are less satisfied with their care. By identifying these groups, initiatives can be developed to address their specific needs. Furthermore, this study incorporates qualitative data to elucidate more about the prenatal care experience for pregnant women in American Samoa.

## Methods

### Setting

American Samoa is an unincorporated US territory and home to a population of 54,194 (2016) [25] predominantly made up of native Samoans who are recognized as US nationals. Fifty-eight percent of families have incomes below the US poverty level [ref]. Health care is provided by one full service hospital – the Lyndon B Johnson Tropical Medical Center (LBJTMC) - and three primary care centers run by the American Samoan Department of Health. Prenatal care is provided at all of these locations. The three primary care centers deliver prenatal care to low-risk pregnancies until 28 weeks gestation using a system of nurse practitioners supported by a clinician at each site. LBJTMC provides care to some low risk pregnancies (particularly those in the immediate geographic area around the hospital), high-risk pregnancies, and all women beyond 28 weeks gestation. At LBJTMC, prenatal care is provided by clinicians with specialist training in obstetrics and gynecology. There are allied health professionals (nutritionists, physical therapists) available but there are few and demand often exceeds capacity. Approximately 97% of the 1300 births in the territory each year occur at LBJTMC. While there is a history of home births attended by traditional birth attendants [26] in the territory, a similar proportion of births have occurred at LBJTMC for more than 15 years.

### Data collection

A 59-question survey targeting utilization, content, and satisfaction with prenatal care was distributed to a convenience sample of patients in the prenatal care clinic at the Lyndon B Johnson Tropical Medical Center, American Samoa between July and August 2014 and again in August 2015. LBJTMC is the only full service hospital in American Samoa, providing prenatal care to some low risk pregnancies (others are seen at community health centers), all high-risk pregnancies, and all women in the final trimester of their pregnancy (women are referred from the community health centers during the third trimester) [14]. The Department of Health managed community health centers include the Tafuna, Amouli and Leone Community Health Centers. Although the survey was only conducted at the LBJTMC, participants were asked to report which of the clinics they attended most frequently during their pregnancy.

The eligibility criteria for participation were that the participants must be over 18 years of age and must have attended at least two prenatal care visits before the visit during which they were enrolled into the study, to allow them to adequately reflect on their experience and their care. Trained study staff approached all women waiting in the prenatal care clinic, explained the purpose and protocol of the study, asked screening questions to gauge eligibility and, if participants were eligible and willing, gained informed consent. The questionnaires were self-administered in the prenatal care clinic waiting room and presented questions in both English and Samoan languages side-by-side to accommodate local language preferences. Participants self-reported their demographic characteristics, receipt of prenatal care, interactions with health professionals, and their satisfaction with prenatal care. The questionnaire was based on the Centers for Disease Control (CDC) Pregnancy Risk Assessment and Monitoring Survey (PRAMS) [27] and the Prenatal Care Satisfaction Questionnaire, which was developed specifically for use in low-income settings [28]. An open-ended comment section was included at the end of the survey to solicit additional participant comments about their experience of prenatal care. The questionnaire instrument can be found in Additional file 1.

Data was collected from 174 participants. 215 patients were approached; of these, 34 did not meet eligibility criteria and seven participants declined to participate. After data collection was complete, one participant was excluded from all analyses because she was determined not to have met eligibility criteria (only discovered after data collection) and a further eight participants were excluded from this analysis because they did not have complete satisfaction questionnaire data, leaving 165 participants for this analysis. The sample represented approximately 8% of the unique prenatal care patients seen by LBJTMC each year.

Institutional review boards at Brown University (Protocol #1405001052) and the American Samoa Department of Health reviewed the study protocol and gave their approval. Participants in this study gave written informed consent.

### Predictor variables

Demographic variables such as age, marital status, resident status, education level, and employment status were included in the analysis as potential predictors of prenatal care satisfaction. Age was categorized into five year age groups between 20 and 36 years, with those 20 years and younger and 36 and older considered separately. Marital status was dichotomized with women either married or cohabiting versus never married, separated, divorced, or widowed. Participants were classified as a resident of American Samoa or a non-resident. Residency status was considered here as non-residents do not have the same access to government services and benefits (WIC services, Medicaid, etc.) as residents. Non-residents in the sample were predominantly from the neighboring island of independent Samoa, which is nearly identical in ethnic background and cultural history. It is common for women in Samoa to travel to American Samoa and stay with extended family during pregnancy and for the birth of their child. Related to this, racial/ethnic background was collected by the survey but because more than 98% of the sample identified themselves as Samoan/Pacific Islander, it was not examined as a predictor of satisfaction. Education level was categorized into secondary school or less versus higher education and women were classified as employed, on maternity leave, unemployed or students.

Maternal characteristics related to pregnancy that were included as predictors of satisfaction included self-reported pre-pregnancy weight, trimester at the time of the survey, pregnancy complications, parity, and prior pregnancy losses. Pre-pregnancy weight was categorized into data-driven tertiles. Body mass index (BMI) could not be calculated due to substantial misreporting of height in the self-reported questionnaire. Parity was categorized based on number of live births. Women who answered yes to a question enquiring about first pregnancy were categorized as nulliparous unless they went on to specify a number of prior live births. The prior pregnancy loss variable was created by subtracting the number pregnancies that resulted in live births from the number of times a woman had been pregnant before this pregnancy.

Structural predictors included distance from the participant’s home to the clinic, health insurance status, WIC enrollment (which is based on residency status and means testing), appointment attendance, waiting time at the clinic and average time spent with clinicians. “Clinic distance” was defined as the driving distance from the participant’s home to their most visited clinic. This was calculated using Google maps (www.maps.google.com). The questions about pregnancy complications, health insurance, WIC enrollment were asked in a yes/no format. Women were also asked to respond yes or no to the question “Did you attend all of the prenatal care visits that were scheduled for you?” Total time spent at the clinic was categorized into 30-min intervals from 0 to more than two hours. The amount of time spent waiting to see the doctor and spent talking with the doctor were asked as open-ended questions and analyzed as continuous variables.

### Outcome variable: Satisfaction with prenatal care

The Prenatal Care Satisfaction Questionnaire developed by Raube et al. [24] comprised 22 of the 59 questions on the survey. Response options for each question were on a Likert scale from Excellent (5) to Poor (1). No reverse scoring was needed due to the structure of the questionnaire.

### Identification of satisfaction domains: Principal components analysis

An exploratory principal components analysis (PCA) was conducted to determine domains of satisfaction that should be considered in analysis. Using the Statistical Program for the Social Sciences (SPSS; Version 22.0, SPSS Inc., Illinois, USA) an initial PCA was conducted on the 22 items with no rotation. Following the initial PCA, one question (How would you rate the explanation of treatment options?) registered a loading of less than 0.30 (the threshold set a priori) and was removed, likely because few participants reported complications for which treatment was required (see Table 1). A second PCA was then conducted on the 21 remaining items with oblique rotation (direct oblimin). The Kaiser-Meyer-Olkin (KMO) measure verified the sampling adequacy for the analysis, KMO = 0.94. Bartlett’s test of sphericity, X^2^ (210) = 3756.19, *p* < 0.001, indicated that correlations between items were sufficiently large for PCA [29]. An initial analysis was run to obtain eigenvalues for each component in the data. Three components had eigenvalues over Kaiser’s criterion of 1 and in combination explained 76.45% of the variance. Given the sufficiency of the sample size and Kaiser’s criterion on the three components, we retained all three components in the final analysis. Table 2 shows the factor loadings after rotation.

**Table 1:** Characteristics of the study sample

Characteristic	N (%)^a^ (Unless Specified)
Age (years) [mean ± SD]	26.7 ± 6.0
20 or younger	27 (16.4)
21–25	55 (33.3)
26–30	42 (25.5)
31–35	26 (15.8)
36 or older	15 (9.1)
Marital Status	
Single (never married, separated, divorced, widowed)	41 (25.0)
Married or Cohabitating	123 (75.0)
Education Level (Highest Attainment)	
Secondary School or Less	74 (45.4)
Higher Education	89 (54.6)
Employment Status	
Unemployed/Student	97 (59.1)
Employed/On Maternity Leave	67 (40.9)
Resident Status	
Resident (Permanent, American Samoa)	129 (79.1)
Non-Resident	34 (20.9)
Health Insurance	
No	131 (84.0)
Yes	25 (16.0)
WIC Enrolled	
No	12 (7.5)
Yes	145 (90.6)
Parity [mean ± SD)	2.7 ± 1.9
Nulliparous	48 (29.8)
1–2 births	64 (39.8)
3–4 births	35 (21.7)
5 or more births	14 (8.7)
Pre-Pregnancy Weight Tertiles [mean ± SD]	85.1 ± 23.6
Tertile 1: 43–72 kg [62.3 ± 8.1]	56 (35.2)
Tertile 2: 73–94 kg [84.4 ± 5.9]	50 (31.4)
Tertile 3: 95–195 kg [109.8 ± 19.5]	53 (33.3)
Number of Prenatal Care Visits Prior to Survey Completion [mean ± SD]	5.9 ± 3.22
Trimester at the Time of Survey	
First	18 (11.8)
Second	48 (31.6)
Third	86 (56.6)
Pregnancy Complications (Current Pregnancy)	
No	146 (89.0)
Yes	18 (11.0)
Previous Pregnancy Loss	
No	97 (82.2)
Yes	21 (17.8)
Most Visited Clinic	
LBJTMC	129 (81.6)
Other	29 (18.4)
Driving Time to Clinic	
Less than 10 min	71 (43.6)
11–19 min	70 (42.9)
20 min or more	22 (13.5)
Average Time Spent at Clinic Per Appointment	
0–30 min	12 (7.3)
30 min – 1 h	50 (30.5)
1 h – 1 h 30 min	24 (14.6)
1 h 30 min – 2 h	25 (15.2)
More than 2 h	53 (32.3)
Average Time Spent Waiting to see a Physician (minutes) [mean ± SD]	54.7 ± 51.4
Average Time Spent Talking to a Physician (minutes) [mean ± SD]	17.8 ± 21.0
Attended All Scheduled Appointments	
No	15 (9.4)
Yes	145 (90.6)

^a^Percentages may not sum to 100 due to rounding

**Table 2:** Dimensions of prenatal care satisfaction based on factor analysis

Dimension	Constructs measured	Factor loading^a^
Clinic Services	How would you rate the *availability of nutritional services* (people who can talk to you about what to eat during pregnancy?)	0.859
	How would you rate the *respect shown to you by the nurses or receptionists*?	0.824
	How would you rate the *comfort shown to you by the nurses or receptionists*?	0.780
	How would you rate the *explanation of procedures*?	0.770
	How would you rate the *helpfulness of advice you have received* from the prenatal clinic during your pregnancy?	0.768
	How would you rate the *thoroughness of your examination*?	0.768
	How would you rate the *explanation of your lab results*?	0.760
	How would you rate the *concern shown to you by the nurses and receptionists*?	0.739
	How would you rate the *availability of doctors*?	0.721
	How would you rate the *cleanliness of the clinic*?	0.709
	How would you rate the *atmosphere of the waiting room*?	0.639
	How would you rate the *comfort of the waiting room*?	0.610
Clinic Accessibility	How would you rate the *waiting time to get an appointment* (between the time you call and come in)?	0.745
	How would you rate the *length of time you wait to see your doctor* when you have an appointment?	0.697
	How would you rate the clinic when thinking about the *hours it is open*?	0.670
	How would you rate the *location of the clinic*?	0.638
Physician’s Interactions	How would you rate the *comfort shown to you by doctors*?	0.900
	How would you rate the *respect shown to you by doctors*?	0.876
	How would you rate the *concern shown to you by doctors*?	0.847
	How would you rate the *technical skills shown to you by the doctors*?	0.745
	How would you rate the *modernness of the medical equipment* in the clinic?	0.399

^a^Factor loadings were extracted using pattern matrix from oblique rotation

Responses to questions within each of the three satisfaction domains were summed to create a satisfaction score for each, and all of the responses summed to create an “overall” satisfaction score. Scores were normalized on a 0–100 scale to account for the different numbers of questions contributing to the scores in each domain and to allow scores to be compared across domains.

### Statistical analysis

The number of participants per group for categorical variables (as percentages) and means for continuous variables were computed for all participants. Based on the normal distribution of outcome variables, independent samples t-tests and ANOVA were used to examine unadjusted differences in mean satisfaction scores among different demographic and characteristic variables.

Multivariable linear regression was used to calculate adjusted mean difference estimates in satisfaction scores using SAS software (Version 9.3; SAS Institute Inc., Cary, NC, USA). Age, parity, resident status, and employment status were kept in all multivariable models regardless of statistical significance at the bivariate level because of their role as key sociodemographic characteristics of the sample. Other sociodemographic variables that were associated with the satisfaction outcomes at a *p* value of 0.1 or less in bivariate analyses were included as categorical covariates in the multivariable regression models. Models were checked for critical assumptions and evaluated for appropriateness according to variance inflation factor, condition index, leverage, and Cook’s distance model diagnostics.

One continuous predictor, time spent talking with the doctor, was analyzed independently against the satisfaction domains using Pearson’s correlations.

### Qualitative analysis

Open-ended patient feedback on the prenatal care experience was solicited at the end of the questionnaire with the statement, “If you would like to provide any other information or comment on your prenatal care experience, please do so here…” Seventy-five participants (43.1%) chose to provide feedback. All comments written in Samoan were translated into English by Samoan research staff. Two of the authors (OA and NH) independently categorized the comments based on positive or negative content. If a comment contained both types of content it was included in both categories. The two reviewers met to reach consensus on the categorization of participant comments.

## Results

The average age of participants in the sample was 26.7 years (Table 1). Over 70% of the participants were married or cohabitating with a partner. Over three quarters of the sample were residents of American Samoa, with non-residents mostly from neighboring Samoa. Approximately 57% of the sample was in their third trimester at the time of the survey, which reflects the general prenatal care population at LBJTMC [8, 14]. Eighty-one percent reported having received all of their care at LBJ and over 90% reported having attended all the appointments scheduled for them. For most women this was not a first pregnancy, with the average parity of the sample being 2.7 births.

The PCA analysis identified three ‘domains’ of satisfaction. The items that clustered within each component suggest that component one represented satisfaction with “clinic services”, component two represented satisfaction with “clinic accessibility”, and component three satisfaction with “physician interactions”. The questions within the “clinic services” component, the “accessibility” component and the “physician interactions” component and overall satisfaction all had high internal consistency with Chronbach’s alphas 0.96, 0.89, 0.93 and 0.97 respectively.

Table 3 presents the normalized mean satisfaction scores for each domain. Mean satisfaction was lowest for “Accessibility” (58.5 points out of 100) and highest in the “Physician Interactions” domain (76.4 points). Overall satisfaction had a normalized mean of 68.4 points out of 100.

**Table 3:** Dimensions of satisfaction: means, ranges and reliability

	Number of questions	Normalized mean	Normalized SD	Normalized range	Standardized Chronbach’s Alpha
Clinic Services	12	68.0	23.9	20–100	0.962
Clinic Accessibility	4	58.5	19.8	20–100	0.892
Physician’s Interactions	5	76.4	20.2	20–100	0.925
Overall Satisfaction	21	68.4	21.9	20–100	0.969

Unadjusted mean satisfaction scores for “clinic services” were associated with participant’s employment status, the driving time to the clinic, and the average time spent at the clinic during each appointment (Table 4). Unadjusted satisfaction with “clinic accessibility” was also associated with driving time to the clinic and the average time spent at the clinic, but also with participant’s resident status. Significant differences in unadjusted mean satisfaction score for “physician interactions” were found with participant employment status and driving time to the clinic. With regards to overall satisfaction, driving time to the clinic, participant employment status, and average waiting time at the clinic were all associated significantly with satisfaction score.

**Table 4:** Unadjusted associations between maternal and clinic characteristics and satisfaction scores

	Clinic services		Clinic accessibility		Physician’s interactions		Overall satisfaction	
Characteristic	Mean	*p* value	Mean	*p* value	Mean	*p* value	Mean	*p* value
Age (years)								
20 or younger	67.7		55.9		77.6		67.8	
21–25	68.1		58.6		75.9		68.1	
26–30	75.0	0.11	64.0	0.11	80.9	0.17	74.3	0.11
31–35	61.3		48.8		68.6		60.7	
36 or older	62.8		63.7		79.2		66.9	
Marital Status								
Single^a^	68.8	0.89	59.5	0.79	76.9	0.96	68.9	0.90
Married/Cohabitating	68.3		58.4		76.8		68.4	
Education Level								
Secondary School or Less	68.6	0.72	58.6	0.83	77.3	0.56	68.8	0.68
Higher Education	67.3		57.8		75.5		67.4	
Employment Status								
Unemployed/Student	70.9	**0.04**	60.8	0.10	79.1	**0.04**	70.9	**0.03**
Employed/Maternity Leave	63.9		54.6		72.6		64.2	
Resident Status^b^								
Resident	67.8	0.68	55.5	**<0.01**	75.1	0.10	67.2	0.19
Non-resident	69.6		69.1		81.5		72.3	
Health Insurance								
No	67.3	0.29	58.8	0.94	75.8	0.37	67.7	0.39
Yes	72.5		58.4		79.7		71.5	
WIC Enrolled^c^								
No	75.1	0.24	70.0	0.09	76.7	0.98	74.5	0.26
Yes	67.4		57.6		76.5		67.7	
Parity								
Nulliparous	68.0		58.5		77.3		68.4	
1–2 births	69.1	0.80	55.7	0.42	77.7	0.82	68.6	0.86
3–4 births	69.3		64.3		75.9		69.9	
5 or more births	62.9		58.6		72.3		64.3	
Pre-Pregnancy Weight^d^								
94–160 lbs	66.5		55.9		73.6		66.2	
161–208 lbs	68.6	0.88	60.5	0.50	76.9	0.60	69.0	0.76
209–430 lbs	66.7		55.6		77.1		67.3	
Trimester at the Time of Survey								
First	71.5		63.1		81.1		72.2	
Second	65.6	0.62	57.0	0.61	73.4	0.36	65.8	0.52
Third	67.4		57.2		76.3		67.6	
Pregnancy Complications								
No	68.0	0.60	58.6	0.88	77.0	0.68	68.3	0.80
Yes	70.8		59.4		74.9		69.6	
Previous Pregnancy Loss								
No	68.7	0.50	58.2	0.99	77.8	0.08	68.9	0.41
Yes	65.2		58.3		69.3		64.9	
Most Visited Clinic								
LBJTMC	68.5	0.38	58.0	0.90	77.0	0.41	68.5	0.48
Other	64.5		58.6		73.7		65.6	
Driving Time to Clinic								
Less than 10 min	72.7		65.3		81.7		73.5	
11–19 min	65.6	**0.02**	54.3	**<0.01**	74.5	**<0.01**	65.6	**<0.01**
20 min or more	59.2		46.4		64.5		58.1	
Average Time Spent at Clinic Per Appointment								
0–30 min	79.0		75.4		85.0			
30 min – 1 h	76.0		67.2		80.1		75.3	
1 h – 1 h 30 min	72.2	**<0.01**	62.3	**<0.01**	77.5	0.13	72.2	**<0.01**
1 h 30 min – 2 h	66.0		56.2		74.1		66.1	
More than 2 h	56.9		45.4		71.8		58.3	
Attended All Scheduled Appointments								
No	58.1	.07	52.3	0.32	70.9	0.26	60.1	0.10
Yes	69.1		58.8		77.0		69.0	

Bolding denotes significance at *p* < 0.05; Binomial variables were analyzed using independent t-tests, categorical variables with ANOVA

^a^Single includes never married, separated, divorced and widowed

^b^Resident status refers to having permanent residence in American Samoa

^c^WIC enrollment may be for the pregnant participant or another child, or both

^d^Weight ranges represent data driven tertiles (see Table 1)

Adjusted linear regression models are presented in Table 5. The “clinic services” satisfaction score was 20.3 points lower among women who waited, on average, two hours or more before they saw the doctor compared with women who waited less than 30 min. Importantly, women who reported not attending all of their appointments were approximately 16 points (95% CI: -28.8, −3.1) less satisfied with “clinic services” than women who did report attending all of their appointments.

**Table 5:** Adjusted associations between maternal and clinic characteristics and satisfaction scores

	Clinic Services Adj. R^2^ = 0.19		Clinic Accessibility Adj. R^2^ = 0.24		Physician’s Interactions Adj. R^2^ = 0.10		Overall Satisfaction Adj. R^2^ = 0.16	
Characteristic	B	95% CI	B	95% CI	B	95% CI	B	95% CI
Age (years)								
20 or younger	−9.4	−21.3, 2.5	−8.3	−20.9, 4.2	−0.4	−16.3, 15.4	−8.4	−19.2, 2.5
21–25	−9.1	−18.4, 0.2	−3.0	−12.8, 6.8	−8.9	−19.1, 1.3	−7.2	−15.8, 1.4
26–30 (ref)	–		–		–		–	
31–35	−6.2	−17.3, 4.8	−7.6	−19.2, 4.0	−10.0	−21.0, 1.1	−6.3	−16.5, 3.8
36 or older	−8.7	−22.3, 4.8	0.2	−13.1, 4.4	−4.7	−18.0, 8.7	−5.0	−17.4, 7.5
Parity								
Nulliparous (ref)	–		–		–		–	
1–2 births	−1.8	−10.2, 6.6	−4.5	−13.4, 4.4	−0.6	−16.7, 15.6	−2.4	−10.1, 5.3
3–4 births	−1.1	−11.6, 9.4	2.6	−8.3, 13.5	1.0	−15.9, 17.8	−0.6	−10.3, 0.1
5 or more births	−6.8	−20.5, 7.0	1.9	−12.6, 16.3	−0.6	−19.3, 18.2	−5.3	−17.9, 7.3
Resident Status^a^								
Resident (ref)	–		–		–		–	
Non-resident	−1.0	−9.3, 7.3	11.7	3.0, 20.5	6.0	−3.5, 15.5	2.4	−5.2, 9.9
Employment Status								
Unemployed/Student (ref)	–		–		–		–	
Employed/Maternity Leave	−6.9	−14.0, 0.2	−2.6	−10.2, 4.7	−9.2	−17.4, −0.9	−5.8	−12.3, 0.7
Driving Time to Clinic								
Less than 10 min (ref)	–		–		-		–	
11–19 min	−1.0	−8.3, 6.3	−5.2	−12.8, 2.5	−0.3	−8.6, 8.1	−2.2	−8.9, 4.5
20 min or more	−8.1	−18.3, 2.0	−13.7	−24.4, −3.0	−14.7	−26.9, −2.5	−10.3	−19.6, −1.0
Average Time Spent at Clinic Per Appointment								
0–30 min (ref)	–		–				–	
30 min – 1 h	−2.3	−16.9, 12.3	−1.1	−16.5, 14.3			−1.8	−15.2, 11.6
1 h – 1 h 30 min	−5.2	−21.1, 10.8	−2.1	−19.1, 14.8			−4.1	−18.7, 10.6
1 h 30 min – 2 h	−12.7	−28.8, 3.4	−9.7	−26.6, 7.3			−10.9	−25.7, 3.8
More than 2 h	−20.3	−35.1, −5.6	−19.8	−35.4, −4.1			−14.1	−25.9, −2.3
Attended All Scheduled Appointments								
No	−16.0	−28.8, −3.1					−14.1	−25.9, −2.3
Yes (ref)	–						–	
WIC Enrolled^b^								
No			−10.6	−23.4, 2.2				
Yes (ref)			–					
Previous Pregnancy Loss								
No (ref)					–			
Yes					−11.2	−21.6, −0.7		

^a^Resident status refers to having permanent residence in American Samoa

^b^WIC enrollment may be for the pregnant participant or another child, or both

Participants living 20 min or further away from the prenatal clinic they most regularly visited were significantly less satisfied with “clinic accessibility”, “physician interactions” and overall, than women who lived less than 10 min from the clinic (satisfaction scores were between 10.3 and 14.7 points lower).

With regard to satisfaction with “clinic accessibility”, non-residents were significantly *more* satisfied compared to American Samoan residents, by 11.7 points (95% CI: 3.0, 20.5). Similar to the findings for other satisfaction domains, women who waited two hours or more were substantially less satisfied compared to those who had waited less than 30 min (difference in means score: 19.8 points; 95% CI: -35.4, −4.1).

Satisfaction with “physician interactions” was negatively associated with employment. Women who were employed/on maternity leave were 9.2 points (95% CI: -17.4, −0.9) less satisfied with their interactions with their physician than women who were unemployed/students. Among only those women who had been pregnant before, women who had previously lost a pregnancy were 11.2 points (95% CI: -21.6, −0.7) less satisfied than those who had never experienced a pregnancy loss.

Overall satisfaction was significantly associated with waiting time at the clinic; women who waited on average two hours or more were 14.1 points less satisfied than women who waited less than 30 min (95% CI: -25.9, −2.3). Pearson’s correlation tests indicated that the average amount of time spent speaking with a physician during prenatal care visits was positively associated with satisfaction with “clinic services” (r^2^ = 0.22, *p* = 0.006), “physician interactions” (r^2^ = 0.19, *p* = 0.017) and overall satisfaction (r^2^ = 0.214, *p* = 0.007) (Table 6).

**Table 6:** Correlation between time spent talking with physicians and satisfaction scores

		Clinic services	Accessibility	Physician interactions	Overall satisfaction
On average, how many minutes do you spent talking with the doctor/being examined?	Pearson Correlation	0.22	0.15	0.19	0.21
	*P*-value	< 0.01	0.06	0.02	< 0.01
	N	157	156	156	156

### Qualitative findings

Of the 75 participant responses to the question “If you would like to provide any other information or comment on your prenatal care experience, please do so here…”, 53 comments contained negative comments, and 29 contained positive comments about the prenatal care experience. Predominant themes among the negative survey comments were long waiting times, limited availability of doctors and nurses, and discomfort in the waiting room. For example, one participant explained, *“I think that the nurses are very kind. My only problem is the waiting area, the waiting time to see the doctors… I feel that regardless of how many patients are waiting to see the doctor, they should try to at least accommodate you and everything you ask”.*

Some patients implied that the long wait times might be due to the limited availability of equipment in the clinic as well as the generally limited availability of doctors and nurses’. One women made this observation when she explained, “… *there should be more ultrasound machines added because that’s probably why pregnant women have to wait for long hours or minutes*”.

Positive comments were general, for example *“the prenatal care program has played an important role in my life as well as other pregnant women. Keep up the good job!”*, and often relayed positive interactions with the nurses and doctors in the clinic or positive feelings about how they approached their tasks: “*I know the nurses’ job and especially the doctor’s job is not an easy task. They try their very best to assist pregnant mothers. A job well done to them…”.* Further comments are included for illustration in Table 7.

**Table 7:** Examples of patient statements about prenatal care

Participant ID	
111 Age 26–30 5th pregnancy	I know the nurses’ job and especially the doctor’s job is not an easy task. They try their very best to assist pregnant mothers. A job well done to them and do continue your usual jobs. Also allow sufficient time for prenatal care visits. Thank you!
174 Age 20 or younger 1st pregnancy	The prenatal care program has been played an important role in my life as well as other pregnant women. Keep up the good job.
233 Age 26–30 1st pregnancy	Prenatal clinic staff is very helpful and polite. I love the nurses. Always kind. My current doc didn’t tell me about many of the things asked in this survey. Equipment needs upgrade and waiting room needs to be expanded. Doctors need to be more involved in a sense and more inclined to ask and test when needed. Also need psychological clinic for pregnant women…
213 Age 26–30 4th pregnancy	My main concern would be the availability of doctors and waiting area. The waiting area needs more room and space.
147 Age 21–25 1st pregnancy	I think that the nurses are very kind. My only problem is the waiting area, the waiting time to see the doctors. I am aware that they have to run errands but when setting an appointment, they should try to stick with the apt. as much as possible. I feel that regardless of how many patients are waiting to see the doctor, they should try to at least accommodate you and everything you ask. I had an experience with one of the doctors that he was rushing me with all the questions I had. I do think that the prenatal program is great and they do take care of us as far as allowing us to be seen for free. Not a lot of hospitals give those services.
257 Age 31–35 2nd pregnancy	Prenatal care is on the average basis. Some receptionists/nurses are caring and comforting, while some are not. We need more doctors and a bigger more comfortable clinic. We need nutritionists at least at our 1st or 2nd visits to talk about nutritional eating or pregnancy diet. Nonetheless, we need a new ultrasound scanner. :)
292 Age 26–30 4th pregnancy	The service is good but the time, the patient wait is so long. Appointment should be on time, as we have things to do. Time is important not really in prenatal but in every section in this hospital.
229 Age 31–35 1st pregnancy	The major issue I have with the clinic is the amount of time it takes to see the Doctor. It takes 2 to 3 h to see a Doctor, regardless of the time of your appointment. If this can be addressed and new procedures for check in are made, I would be happy with the service.
218 Age 21–25 1st pregnancy	The service provided by the doctors and nurses at the clinic is outstanding but the only problem is that the clinic is too small and the waiting room does not have enough space for all pregnant women coming to the clinic for their prenatal visits.
101 Age 26–30 2nd pregnancy	It’ll be nice if we are provided with information on prenatal clinics and pregnancy especially some women are new at it. Although this is my second baby there are still more information I would like to know in order for me to be prepared for my future pregnancies such as weight loss/gain before, during, and after pregnancy.

## Discussion

Our results confirm that specific maternal characteristics are associated with prenatal care satisfaction overall and with specific domains of satisfaction. These characteristics go beyond basic demographic traits and take into account the social and environmental characteristics of women’s prenatal care experiences. Clinic distance, specifically living more than 20 min driving time from the clinic most frequently attended, was significantly associated with lower satisfaction with “clinic accessibility”, “physician interactions” and overall satisfaction. Being employed or on maternity leave was significantly associated with lower satisfaction with “physician interactions” and approached significance in the same direction in the “clinic services” and overall satisfaction domains. Average time spent at the clinic for a prenatal care appointment, particularly spending two hours or more at the clinic, was associated with “clinic accessibility”, “clinic services” and overall satisfaction. Previous pregnancy loss emerged as a pertinent predictor of lower satisfaction with “physician interactions”. Non-resident status was the only positive predictor of satisfaction with higher satisfaction among non-residents than residents with “clinic accessibility”.

Other studies of satisfaction with prenatal care have reported similar findings. In Ethiopia, Chemir et al. [20] found that dissatisfaction with prenatal care was primarily due to long waiting times, overcrowding in the clinic during the morning, and poor laboratory services. Handler et al. [21] suggested that, to improve satisfaction, prenatal care providers should focus on improving provider-patient communication, clinic cleanliness, waiting times, and availability of ancillary services based on their study of low-income pregnant women in the US.

The time required to attend prenatal care seemed to be a key determinant of satisfaction among participants in this study: time spent traveling to the clinic, waiting time at the clinic, the amount of time spent with the physician, and time taken away from employment to attend appointments. The “clinic accessibility” domain, which reflected satisfaction with clinic location, waiting times, and opening hours, received the lowest mean satisfaction scores from study participants. The distance between a participant’s home village and the clinic they used most frequently was a strong predictor of satisfaction with “clinic accessibility”. Although the island of Tuituila, where this study was conducted, is small, most people rely on public transportation, so a driving distance of 20 min to get to a prenatal care appointment may require a much greater time investment, depending on bus schedules or availability of family to offer transport. The finding that distance to a clinic was associated with “physician interactions” may reflect those who traveled further feeling that they got less ‘value’ for their travel time investment for a comparatively short time spent with the physician (~18 min on average) than women who had a much shorter travel time. Indeed, the distance between a patient’s home and their prenatal care provider has been consistently demonstrated to be a key factor in both satisfaction with prenatal care and prenatal care utilization in other settings [30].

In spite of general dissatisfaction with clinic accessibility, being a non-resident was associated with greater satisfaction in this domain. The majority of non-residents who completed the survey reported being from neighboring Samoa. Birth tourism is common between Samoa and American Samoa; it may be that, given its association with the United States, American Samoa is perceived by women as having more resources for prenatal care than Samoa. Extended families are often spread across both islands and travel between the two is relatively inexpensive by plane or boat so many women from Samoa stay with family in American Samoa for the months immediately preceding their birth. They often choose to stay with family close to the hospital and are ineligible for employment during their stay, as such, they may have found accessing prenatal care less difficult than the American Samoan residents.

In 2009, the Department of Health opened community health centers in Amouli and Leone to expand access to care to residents in the Eastern and Western districts of American Samoa [14]. However, women are only able to attend these clinics until 28 weeks, after which they must go to LBJTMC for their final trimester care. This requirement, although designed with patient safety in mind, may be undermining satisfaction with prenatal care. If further, specialist, staffing was a possibility for these clinics, or if low-risk women could remain at their closest clinic until the time of birth under the care of midwives or qualified nurse practitioners, satisfaction may be improved.

Understandably, the average amount of time spent at the clinic per appointment was a strong predictor of satisfaction in all but the “physician interactions” domain. While satisfaction declined with increasing time spent at the clinic, only those who reported spending more than two hours at the clinic were significantly less satisfied than those who waited for half an hour or less. On average, women waited 57 min after check in at the clinic to meet with a doctor, so it may be that an appointment taking up to 90 min was expected and anything beyond that was unusual and possibly frustrating. Long waiting times are a well-known deterrent to seeking and remaining in prenatal care globally [31–33]. The costs associated with long clinic wait times (lost wages, lost school time, and the costs of childcare for other children) can be burdensome over the course of pregnancy, particularly in low income settings such as American Samoa. Additionally, several studies have shown that women may consider long waiting times for prenatal care (or other healthcare services more generally) to indicate a disrespect by providers for the importance of their time, and that a short consultation with the doctor after a long wait time is particularly insulting [34, 35]. The fact that those women who were employed were less satisfied with “physician interactions” than those who were students or unemployed may indicate that employed women felt particularly strongly that physicians were not respecting their time. While the first-come-first-served system of appointment booking currently operated in American Samoa, with all women given the same early morning appointment time, may be efficient from the clinicians perspective (to avoid wasted time associated with missed appointments) an alternative model could be considered to improve patient satisfaction. To accommodate working mothers, clinic operating hours could be more flexible, perhaps with late/early opening offered on specific days to improve access to care for those women who find it hard to attend during regular working hours.

An additional predictor of satisfaction with physician interactions was previous pregnancy loss. Previous pregnancy loss has been demonstrated to have a profound impact on how a woman navigates and experiences health care [36]. Multiple investigators have documented increased symptoms of depression and pregnancy-related anxiety in pregnant women following a prior miscarriage or stillbirth [37]. With physician interactions so limited in American Samoa it may not be possible to provide the level of support and reassurance necessary to ensure satisfaction with and confidence in prenatal care. Within the “physician interactions” domain, participants ratings of the specific items “How would you rate the *comfort shown to you by doctors*?” and “How would you rate the *concern shown to you by doctors*?” were lower if they had experienced a prior pregnancy loss than if they had not, while there was no significant difference in responses to the other questions in the domain (data not shown). While utilization of care did not differ in this study based on prior pregnancy loss, it may be important to consider providing additional support services to these women outside of the physician interactions that could assist with building confidence in care.

While this was a cross-sectional study, and we cannot prospectively connect satisfaction with prenatal care with utilization, it was evident that those who had not attended all of their scheduled appointments prior to the survey were less satisfied with care – overall and in the “clinic services” domain – than those who had attended all of their scheduled appointments, indicating that satisfaction and utilization may have been linked. Satisfaction in general was lower than reported by Raube et al. [28] in the sample of low-income, US women whose data was used to create the measure of satisfaction used here (overall satisfaction was 73.8 in the original study versus 68.4 here) and lower than that reported among African American women in a managed care organization in the US Midwest (overall satisfaction: 80.3) [24]. This suggests that further attention should be paid to monitoring and improving satisfaction in this setting, to ensure continued attendance at the individual and community level. Our data suggest that efforts to this effect should be focused on improving organizational aspects of care in particular: improving waiting times and increasing accessibility.

Several studies have examined alternative organizational models of prenatal care and measured satisfaction with care as an outcome. Randomized controlled trials in the UK [38], the US [39] and Zimbabwe [40] compared fewer, more objectively focused prenatal care visits with standard of care. Fewer visits should reduce the cumulative time spent traveling to the clinic and waiting, but satisfaction with these programs varied; satisfaction was increased in patients assigned to fewer visits in Zimbabwe and the US, but British patients reported lower satisfaction and more anxiety about pregnancy progress. Advancing this literature, a recent study in California tested a new model of care (OB Nest) and compared fewer prenatal care visits supported by virtual connections (8 office visits plus 6 telephone or online nurse visits) to twelve standard of care office visits [41] and preliminary results suggested that the program improved patient satisfaction (OB Nest = 95% versus usual care = 77%, *p* < 0.001) [42]. In very limited resource settings, reducing prenatal care visits has been associated with increased perinatal mortality [43] and American Samoa may not be equipped to facilitate virtual monitoring, so it would be hard to advocate for either of these as an option for improving prenatal care delivery in this setting. One option that may be feasible is group prenatal care. There is a building literature to suggest that nurse/midwife led prenatal care and education, delivered via facilitated discussion to groups of 8–12 women simultaneously, is associated with large improvements in satisfaction, utilization and pregnancy outcomes [23, 44, 45]. In American Samoa offering group prenatal care to ‘healthy’ pregnancies (those with no complications other than overweight) may improve satisfaction and also reduce burden on the practicing physicians, potentially reducing waiting times for others.

There are some limitations to our study. While we used a satisfaction questionnaire that had been previously validated for use in other settings [28] we only identified three specific domains of satisfaction compared to the seven that were extracted in the initial questionnaire development. The reasons why the questionnaire produced fewer domains requires further investigation. Our domains did demonstrate high reliability and good construct validity, but the fact that our final adjusted models explained between 10 and 24% of variation in satisfaction does suggest that there are other aspects of satisfaction that were not captured by the questionnaire. Further qualitative work may be necessary to understand women’s expectations of care and their satisfaction with it. Perhaps most pertinent of the limitations was that the questionnaire was limited in terms of its ability to explore cultural, social or linguistic factors that may be influencing women’s engagement with prenatal care. There is evidence that cultural beliefs about pregnancy and the appropriate provider of prenatal care may influence timing of initiation of care or the type of care Samoan women seek [26], but how these beliefs may influence satisfaction once a patient has enrolled in clinical care is still to be explored. Reports from neighboring independent Samoa have described ‘clashes’ between Westernized medicine and traditional birthing practices which prioritize women’s “social, cultural, and emotional safety” over their “biological safety” [26] but these issues have been little explored in American Samoa.

Similarly, language barriers between patient and provider have been associated with lower satisfaction with care [46]. Prenatal care clinicians in American Samoa are often from outside of the culture and are unfamiliar with the local language. The effect of provider ethnicity/primary language on satisfaction with care should be further explored in new research.

A further limitation was the self-report nature of the questionnaire, which may have introduced bias. Two pertinent issues arose: a lack of detailed information about the severity of pregnancy complications, which may have affected reports of satisfaction and the frequent misreporting of height (implausibility of values) which made it impossible to examine whether overweight or obesity impacted satisfaction or utilization. However, while weight status has been associated with differential prenatal care utilization in other settings [7, 47] we suspect that it is unlikely to be an issue in American Samoa since more than 90% of women are overweight or obese upon conception [8] and there is little stigma attached to pregnancy body size in this setting [48]. A significant strength to our study was the fact that we surveyed women during pregnancy, as opposed to many studies who have asked about satisfaction retrospectively, meaning that we may expect little bias based on birth experiences.

## Conclusions

Prenatal care satisfaction is an important determinant of prenatal care utilization and in American Samoa, where many pregnancies are high risk, ensuring adequate utilization of prenatal care services is imperative. This study suggests that there is substantial room for improvement in pregnant women’s satisfaction with prenatal care in American Samoa, and that efforts to improve satisfaction should focus on improving waiting times and clinic accessibility. Further research is necessary to explore other social and cultural factors that may be impacting satisfaction with care and preventing optimal utilization of prenatal care services.

## Additional file

Additional file 1: Prenatal care questionnaire. Questionnaire instrument used to collect data on prenatal care satisfaction. (PDF 125 kb)

## Abbreviations

BMI: Body mass index
CDC: Centers for disease control
CI: Confidence interval
LBJTMC: Lyndon b johnson tropical medical center
PCA: Principal components analysis
PRAMS: Pregnancy risk assessment and monitoring survey
WIC: Women, infants and children
