# Supplementary material for: Predictors of prenatal care satisfaction among pregnant women in American Samoa
Source: BMC Pregnancy Childbirth. 2017 Nov 16;17:381. doi: 10.1186/s12884-017-1563-6 (PMC5689158; doi:10.1186/s12884-017-1563-6)
Supplement: Additional file 1: — Prenatal care questionnaire. Questionnaire instrument used to collect data on prenatal care satisfaction. (PDF 125 kb) [file 12884_2017_1563_MOESM1_ESM.pdf]

About You (*Fa'atatau ia oe*)

1. How old are you? (*O le a lou matua?)* ______Years(*Tausaga)*
2. What is your marital status? (*O le a lou tulaga tau fa'aipoipoga?)*

   Never married (*E le'i fa'aipoipo)* (0) ☐ Currently married (*O lo'o fa'aipoipo i le taimi nei)* (1) ☐

   Separated (*E le toe nonofo fa'atasi*) (2) ☐ Divorced (*Tete'a*) (3) ☐

   Widowed (*Maliu lou to'alua*) (4) ☐ Living with a partner (*Nonofo ma se paaga)* (5) ☐

3. What village do you live in (*O fea le alalafaga e te nofo ai?)* ____________________________

4. Are you a resident? (*O fea le atunu'u o e nofo i ai?)*

   American Samoa (*Amerika Samoa*) (0) ☐ Western Samoa (*Samoa i Sisifo)* (1) ☐

   Other (*Se tasi atunu'u)* (please write where) (*Fa'amolemole fa'amaoti mai)* (2) ☐
   ____

5. What is your ethnicity? (*O oe o se tagata nu'u o i fea le atunu'u?)*
   ____

6. What is the highest level of education you have received?*(O le a le maualuga o le a'oga na e mauaina?)*

   No formal schooling (*E le'i a'oga*) (0) ☐

   Less than primary school (*E le'i ma'ea le a'oga tulaga muamua*) (1) ☐

   Primary school completed (*Ua mae'a le aoga tulaga muamua*) (2) ☐

   Secondary school completed (*Ua ma'ea le aoga tulaga lua*) (3) ☐

   College / University completed (*Ua ma'ea le kolisi tu'ufa'atasi po'o le univesite*) (4) ☐

   Postgraduate degree (*Aoga o le Masters po'o le fa'afoma'i*) (5) ☐

7. How many <u>years</u> of education have you received?(*E fia <u>tausaga</u> na e aoga ai?)* ____ Years *(Tausaga)*

8. Are you currently employed? (*O e faigaluega i le taimi nei*?)

   No (*Leai*) (0) ☐ Yes, Full Time (*Ioe, Taimi Atoa*) (1) ☐

   Yes, Part Time (*Ioe, Se Taimi Pu'upu'u*) (2) ☐ Student (*Tamaititi Aoga*) (3) ☐

   On maternity leave *(Livi tinā ma'itaga*) (4) ☐

9. What is your job title? (*O le a le igoa o lou galuega*?)
   ____

10. What was your weight before you got pregnant?(*E fia ou pauna ae ete le'i ma'itaga*?) _______Pounds(*Pauna*)

11. What is your height (*O le a lou umi*?) (please tell us in feet and inches) (*Fa'amolemole ta'u mai i futu ma inisi*)______________________

12. How many weeks pregnant are you (*E fia vaiaso o lou ma'itaga?)* _________weeks (*vaiaso*)

13. Have you experienced any pregnancy complications (problems with your health or your baby's health during this pregnancy? (*Na i ai ni fa'afitauli a'o e ma'itaga (fa'afitauli o lou soifua ma le soifua o lou pepe i lenei ma'itaga?)*

No (*Leai)* (0) ☐ Yes (*Ioe)* (1) ☐

14. Which prenatal care clinic have you attended for most of your visits? (*O fea le potu talavai o tinā ma'itaga e tele na alu ai mo ou talavai ma'itaga?)*

LBJ (0) ☐ Other (*Se isi potu talavai*) (1) ☐

Please specify (*Fa'amolemole fa'amaoti mai*) ______________________________

15. Is this your first pregnancy? (*O lou ma'itaga muamua lenei?)* No(*Leai*) (0) ☐ Yes (*Ioe*) (1) ☐ **(if Yes, go to Question 18) (*Afai e Ioe, fa'auaau atu i le fesili 18*)**

16. How many times have you been pregnant before (*E fa'afia ona e ma'itaga muamua*?) ____ times (*taimi*)

17. How many of those pregnancies resulted in a live birth?(*E fia o nei ma'itaga na soifua*?)

________ live births (*fanau soifua*)

About Your Prenatal Care (*E fa'atatau i le tausiga o tinā ma'itaga)*

18. Are you enrolled in the LBJ Prenatal Program?(*O e auai i le porokalama o tinā ma'itaga o le LBJ*?)
    No (*Leai)* (0) ☐ Yes (*Ioe)* (1) ☐

19. Are you covered by any kind of health insurance or Medicaid? (*O kavaina oe e se inisiua o le soifua po'o le Medicaid?)*
    No (*Leai*) (0) ☐ Yes(*Ioe*) (1) ☐

20. In the past month were you enrolled in the WIC program or did you get WIC vouchers for yourself or for any of your children?(*I le masina ua tuana'i na e auai i le porokalama o le WIC pe na maua mai ni au pepa taumafa mai le WIC mo oe po'o o lau fanau?)* **(Check all boxes that apply) (*Siaki uma pusa o lo'o apalai)***

    No (*Leai*) (0) ☐
    Yes, I was enrolled in WIC myself (*Ioe, na ou auai i le porokalama o le WIC*) (1) ☐
    Yes, my child was enrolled *(Ioe, o lo'o auai lo'u alo i le porokalama o le WIC* (2) ☐

21. How many weeks pregnant were you when you came for your first prenatal visit? (*E fia vaiaso o lou ma'itaga na e sūsū mai ai mo lou ulua'i talavai mo tinā ma'itaga?)*
    _________ weeks(*Vaiaso*)
22. How many prenatal care visits have you attended during this pregnancy (including this one)? *(E fia talavai o tinā ma'itaga na e auai ai i lenei ma'itaga?* (*E aofia ai ma lenei talavai*)
    __________ visits(*talavai*)

23. Have you attended all of the appointments that were scheduled for you? (*Na e auai i talavai uma na fa'atulagaina mo oe?)*
    Yes, I attended all my appointments (*Ioe, Na ou auai i talavai uma na fa'atulagaina*) (0) ☐
    No, I missed at least one appointment (*Leai, Na misi e a'u se talavai fa'atulagaina e tasi*) (1) ☐

24. Did you get prenatal care as early in your pregnancy as you wanted? (*Na e mauaina le tausiga o tinā ma'itaga i le amataga o lou ma'itaga?*)
    No(*Leai*) (0) ☐ Yes(*Ioe*) (1) ☐

25. Did any of these things keep you from getting prenatal care as early as you wanted?
E i ai nisi o mea o lo'o lisi ifo i lalo, e taofia ai lou auai atu i talavai o tinā ma'itaga?

For each item, check T (true) if it was a reason you did not get prenatal care when you wanted or check F (false) if it was not a reason for you or if something does not apply to you
Mo fa'aupuga o i lalo ifo, siaki le 'moni' pe a i ai se mafuaga ua e le auai ai i talavai o tinā ma'itaga pe siaki le 'le moni' pe a leai se mafuaga e apalai ia te oe.

	**True** ***Moni*** **(1)**	**False** ***Le moni*** **(0)**
a. I couldn't get an appointment when I wanted one............................ (*Ua le mafai ona ou mauaina se talavai fa'atulagaina na ou mana'o i ai.)*	☐	☐
b. I didn't have enough money or insurance to pay for my visits.......... (*E le'i lava so'u tupe po'o se inisiua e totogi mo lo'u talavai)*	☐	☐
c. The co-pays on my insurance were too high.................................... (*Ua maualuga tele le peimeni o lo'u inisiua*)	☐	☐
d. I had no transportation to get to the clinic or doctors office.............. (*E leai so'u auala i le potu talavai po'o le ofisa o le foma'i*)	☐	☐
e. The doctor or my health plan would not start care as early as I wanted (*Ua le mafai e lo'u foma'i ona amata le tausiga i se taimi vave na ou mana'o ai)*	☐	☐
f. I had too many other things going on... (*E tele nisi mea na ou pisi i ai.)*	☐	☐
g. I couldn't take time off from work or school....................................... (*E le'i mafai ona fa'aavanoa so'u taimi mai le galuega po'o le aoga*)	☐	☐
h. I didn't have my Medicaid (or state Medicaid Name) card................ (*E leai so'u card o le Medicaid)*	☐	☐
i. I had no one to take care of my children.. (*E leai se isi e tu'u ai lo'u fanau.)*	☐	☐
j. I didn't know that I was pregnant... (*Ou te le iloa ua ou ma'itaga*.)	☐	☐
k. I didn't want anyone else to know I was pregnant.............................. (*Ou te le'i mana'o e iloa e se tasi ua ou ma'itaga.)*	☐	☐
l. I didn't want prenatal care... (*Ou te le'i mana'o e fai so'u talavai mo tinā ma'itaga*.)	☐	☐

26. During your first or second visit to the prenatal clinic did you have any of the following?
(*I le taimi na fai ai lou talavai muamua po'o le lona lua i le potu talavai o tinā ma'itaga, na fa'ataunu'u ni suega o i lalo ifo?)*

	**Yes (1)** ***Ioe***	**No (0)** ***Leai***	**Don't Know (2)** ***Le iloa***
a. A pregnancy test... *Su'ega o le ma'itaga*	☐	☐	☐

	Yes (1) *Ioe*	No (0) *Leai*	Don't Know (2) *Le iloa*
b. A blood pressure measurement.................................... *O le fuaina o le toto*	☐	☐	☐
c. A urine test.. *Suega o le feau vai*	☐	☐	☐
d. A blood test.. *Su'ega o le toto*	☐	☐	☐
e. Your weight measured.. *Fua o le mamafa*	☐	☐	☐
f. Your height measured.. *Fua o le umi*	☐	☐	☐
g. A physical/pelvic examination...................................... *Su'ega o le tino*	☐	☐	☐
h. A health history.. *Fa'amaumauga o lou soifua maloloina*	☐	☐	☐
i. An ultrasound scan... *Su'ega o le fa'aata*	☐	☐	☐
j. A blood glucose test... *O le suega o le suka*	☐	☐	☐
k. An HIV test.. *O le suega o le HIV*	☐	☐	☐

27. During your prenatal care visits did a doctor, nurse, or other healthcare worker talk with you about any of the things listed below?
(*Ina ua fai lau talavai o tinā ma'itaga, na talanoa atu se foma'i, 'au tausi ma'i po'o le aufaigaluega e fa'atatau i mea oi lalo ifo*?)
Please count only discussions, not reading materials or videos. For each item, check Y (yes) if someone talked with you about it or check N (no) if no one talked with you about it. *Fa'amolemole, faitau na'o fa'amatalaga ae le o pepa faitau ma videos. Siaki le 'ioe' pe afai na talanoa atu se tasi e fa'atatau i nei mea pe siaki le 'leai' pe a le talanoaina.*

	Yes (1) *Ioe*	No (0) *Leai*
a. What care would be provided to you by the clinic............................... (*O le a le tausiga o le a e mauaina i le potu talavai.*)	☐	☐
b. The laboratory tests that would be performed.................................... (*O su'ega o le potu tuitoto o le a faia*)	☐	☐
c. What to expect during your pregnancy... (*O mea e ao ina tutupu a'o e ma'itaga.*)	☐	☐
d. Signs and symptoms to be reported to the doctor (e.g. bleeding etc.). *(O ni saini po'o ni fa'aaliga e ripoti i le foma'i (piliiti ma nisi mea*).	☐	☐

	Yes (1) ***Ioe***	No (0) ***Leai***
e. The anticipated schedule of your prenatal care visits......................... (*O se taimi fa'atulagaina mo ou talavai o tinā ma'itaga.)*	☐	☐
f. The presence of doctors during your labor and delivery...................... (*O le auai o foma'i i le fa'afanau ma le fanauga o lo'u pepe*.)	☐	☐
g. The cost of your prenatal care and delivery (e.g. insurance etc.)........ (*O le tau o ou talavai o tinā ma'itaga ma le fanauga o lo'u pepe. (insiua ma nisi mea*)	☐	☐
h. Educational programs available to you... (*O porokalama e aoga mo oe*.)	☐	☐
i. Options for care during your delivery.. (*O le tausiga o oe i le fanauga o lou pepe*.)	☐	☐

28. During your prenatal care visits did a doctor, nurse, or other healthcare worker talk with you about any of the things listed below?
(*Ina ua fai lau talavai o tinā ma'itaga, na talanoa atu se foma'i, 'au tausi ma'i po'o le aufaigaluega e fa'atatau i mea oi lalo ifo*?)
Please count only discussions, not reading materials or videos. For each item, check Y (yes) if someone talked with you about it or check N (no) if no one talked with you about it. *Fa'amolemole, faitau na'o fa'amatalaga ae le o pepa faitau ma videos. Siaki le 'ioe' pe afai na talanoa atu se tasi e fa'atatau i nei mea pe siaki le 'leai' pe a le talanoaina.*

	Yes (1) ***Ioe***	No (0) ***Leai***
a. How smoking in pregnancy could affect your baby............................. (*E fa'apefea ona a'afia lou pepe pe a e taumafa tapa'a a'o ma'itaga*)	☐	☐
b. Breastfeeding your baby... (*O le fa'asusuina o lou pepe i lou suasusu*)	☐	☐
c. How drinking alcohol in pregnancy could affect my baby................... *(E fa'apefea ona a'afia lou pepe pe a taumafa i le ava malosi a'o ma'itaga)*	☐	☐
d. Using a seat belt during your pregnancy... (*O le fa'aogaina o le fusipa'u o le ta'avale a'o e ma'itaga)*	☐	☐
e. Medicines that are safe to use during your pregnancy....................... (*O fualaau po'o vailaau togafiti o lo'o saogalemu e fa'aogaina a'o e ma'itaga)*	☐	☐
f. How using illegal drugs could affect your baby................................... *(Pe fa'apefea ona a'afia lou pepe pe a e fa'aogaina fualaau fa'asaina)*	☐	☐
g. Doing tests for birth defects or diseases that run in your family.......... (*O su'ega e su'e ai ni ma'i i totonu o lou aiga*.)	☐	☐
h. The signs and symptoms of preterm labor.. (labor more than 3 weeks before the baby is due) *(O fa'aaliga o le a toetiti fanau lo'u pepe (pe tusa lea ma le 3 vaiaso o le aso ua fa'atulagaina e fanau ai lou pepe*.)	☐	☐

	Yes (1) *Ioe*	No (0) *Leai*
i. What to do if your labor starts early.. (*O mea e fai pe a fai e tutupu nei fa'aaliga*)	☐	☐
j. Getting tested for HIV (the virus that causes AIDS)............................. (*O su'ega o le HIV*)	☐	☐
k. What to do if you feel depressed during your pregnancy.................. (*O mea e fai pe a le fiafia a o e ma'itaga*)	☐	☐
l. Physical abuse to women by their partners / husbands.................... (*O le sauaina o tinā e a latou paaga po'o to'alua*.)	☐	☐

29. During your prenatal care visits did a doctor, nurse, or other healthcare worker talk with you about any of the things listed below?
Please count only discussions, not reading materials or videos. For each item, check Y (yes) if someone talked with you about it or check N (no) if no one talked with you about it.(Fa'amolemole, faitau na'o fa'amatalaga e le aofia ai video. Siaki le 'ioe' pe afai na talanoa atu se tasi e fa'atatau i nei mea pe siaki le 'leai' pe a le talanoaina.)

	Yes (1) *Ioe*	No (0) *Leai*
a. Foods that are good to eat during pregnancy.................................... *(O taumafa e aoga a'o e ma'itaga*)	☐	☐
b. Taking multivitamins or prenatal vitamins during pregnancy............. (*O le inuina o fualaau vaitamini a'o ma'itaga*.)	☐	☐
c. Taking folic acid during pregnancy... (*O le inuina o fualaau o le folic acid a o ma'itaga*.)	☐	☐
d. How much weight to gain during pregnancy...................................... (*E fia pauna e fa'aopopo a'o e ma'itaga*.)	☐	☐
e. Exercise during pregnancy... (*O le fa'amalosi tino a o ma'itaga*).	☐	☐
f. Programs or resources to help me gain the right amount of weight during pregnancy.. (*O porokalama e fesoasoani ia te oe i le fa'aopopoina o pauna sa'o mo lo'u ma'itaga.)*	☐	☐
g. Programs or resources to help me lose weight after pregnancy......... (*O porokalama e fesoasoani ia te oe e fa'alusi ai ou pauna pe a mae'a lou ma'itaga*.)	☐	☐

30. If a doctor talked to you about weight gain during pregnancy, how much weight did they advise you to gain from the beginning to the end of this pregnancy?
(Afai na talanoa atu se foma'i ia te oe e fa'atatau i pauna fa'aopopo a'o ma'itaga, e fia ni pauna na fautua atu ai le foma'i e fa'aopopo mai le amataga se'i o'o atu i le taimi mulimuli o lou ma'itaga.)
_______pounds (pauna) **OR (PE)**
_____My doctor did not talk to me about weight gain (*E le'i talanoa mai lo'u foma'i e fa'aopopo nisi o'u pauna.*

31. How much weight do you think you SHOULD gain during this pregnancy? ___________ pounds
(E fia ni pauna e te iloa e TATAU ona e fa'aopopoina i lenei ma'itaga? *Pauna*

32. How much weight do you think you WILL gain during this pregnancy? ___________ pounds
    (E fia ni pauna O LE A E fa'aopopoina i lenei ma'itaga?) *pauna*

Prenatal Care Satisfaction (*O lou malie i le tausiga o tinā ma'itaga*.)

The following questions are about your satisfaction with the prenatal care you have received during your pregnancy. Your answers to these questions will be kept confidential. Your answers will not be shared with the doctors and nurses in the prenatal clinic and they will not affect the care you receive at the prenatal clinic. *(O nei fesili e fa'atatau i lou malie i le tausiga o tinā ma'itaga na e mauaina a o e ma'itaga. O tali o nei fesili o le a puipuia malu. O le a le fa'asoa atu fo'i ou tali i foma'i ma le 'au tausi ma'i o le potu talavai o tinā ma'itaga ma o le a le afaina ai fo'i le tausiga o lo'o e mauaina i le potu talavai o tinā ma'itaga.)*

33. How would you rate the respect shown to you by the nurses or receptionists? (*E fa'apefea ona e togiina le fa'aaloalo o lo'o fa'alia e le 'au tausi ma'i ma le aufaigaluega ia te oe?)*

Excellent (5) ☐ Very Good (4) ☐ Good (3) ☐ Fair (2) ☐ Poor (1) ☐
Lelei atoatoa *Lelei tele* *Lelei* *Lelei feololo* *Le lelei*

34. How would you rate the concern shown to you by the nurses or receptionists? (*E fa'apefea ona e togiina le popole o le 'au tausi ma le aufaigaluega mo oe?)*

Excellent (5) ☐ Very Good (4) ☐ Good (3) ☐ Fair (2) ☐ Poor (1) ☐
Lelei atoatoa *Lelei tele* *Lelei* *Lelei feololo* *Le lelei*

35. How would you rate the comfort shown to you by the nurses or receptionists? (*E fa'apefea ona e togiina le faigofie ona e fa'amasani i le 'au tausi ma'i ma le aufaigaluega ia te oe?)*

Excellent (5) ☐ Very Good (4) ☐ Good (3) ☐ Fair (2) ☐ Poor (1) ☐
Lelei atoatoa *Lelei tele* *Lelei* *Lelei feololo* *Le lelei*

36. How would you rate the respect shown to you by the doctors? (*E fa'apefea ona e togiina le fa'aaloalo o lo'o fa'alia e foma'i ia te oe?)*

Excellent (5) ☐ Very Good (4) ☐ Good (3) ☐ Fair (2) ☐ Poor (1) ☐
Lelei atoatoa *Lelei tele* *Lelei* *Lelei feololo* *Le lelei*

37. How would you rate the concern shown to you by the doctors? (*E fa'apefea ona e togiina le popole o le foma'i ia te oe*?)

Excellent (5) ☐ Very Good (4) ☐ Good (3) ☐ Fair (2) ☐ Poor (1) ☐
Lelei atoatoa *Lelei tele* *Lelei* *Lelei feololo* *Le lelei*

38. How would you rate the comfort shown to you by the doctors? (*E fa'apefea ona e togiina le faigofie ona fa'amasani le foma'i ia te oe?)*

Excellent (5) ☐ Very Good (4) ☐ Good (3) ☐ Fair (2) ☐ Poor (1) ☐
Lelei atoatoa *Lelei tele* *Lelei* *Lelei feololo* *Le lelei*

39. How would you rate the modernness of the medical equipment in the clinic? (*E fa'apefea ona e togiina le lelei o masini o lo'o fa'aogaina e le potu talavai*?)

Excellent (5) ☐ Very Good (4) ☐ Good (3) ☐ Fair (2) ☐ Poor (1) ☐
Lelei atoatoa *Lelei tele* *Lelei* *Lelei feololo* *Le lelei*

40. How would you rate the technical skills of the doctor? (*E fa'apefea ona e togiina le tomai o le foma'i?)*

Excellent (5) ☐ Very Good (4) ☐ Good (3) ☐ Fair (2) ☐ Poor (1) ☐
Lelei atoatoa *Lelei tele* *Lelei* *Lelei feololo* *Le lelei*

41. How would you rate the thoroughness of your examinations? (*E fa'apefea ona e togiina le mae'ae'a o ou suega?)*

Excellent (5) ☐ Very Good (4) ☐ Good (3) ☐ Fair (2) ☐ Poor (1) ☐
Lelei atoatoa *Lelei tele* *Lelei* *Lelei feololo* *Le lelei*

42. How would you rate the explanation of procedures? (*E fa'apefea ona e togiina le fa'amatalaina o mea o le a faia ?)*

Excellent (5) ☐ Very Good (4) ☐ Good (3) ☐ Fair (2) ☐ Poor (1) ☐
Lelei atoatoa *Lelei tele* *Lelei* *Lelei feololo* *Le lelei*

43. How would you rate the explanation of your lab results? (*E fa'apefea ona e togiina le fa'amatalaina o ou su'ega i le potu tuitoto*?)

Excellent (5) ☐ Very Good (4) ☐ Good (3) ☐ Fair (2) ☐ Poor (1) ☐
Lelei atoatoa *Lelei tele* *Lelei* *Lelei feololo* *Le lelei*

44. How would you rate the explanation of treatment options? (**if applicable**) (E fa'apefea ona e togiina le fa'amatalaga o togafiti mo oe?)

Excellent (5) ☐ Very Good (4) ☐ Good (3) ☐ Fair (2) ☐ Poor (1) ☐
Lelei atoatoa *Lelei tele* *Lelei* *Lelei feololo* *Le lelei*

This does not apply to me (98) ☐
E le apalai nei mea ia te a'u.

45. How would you rate the cleanliness of the clinic? (*E fa'apefea ona e togiina le mamā o le potu talavai*?)

Excellent (5) ☐ Very Good (4) ☐ Good (3) ☐ Fair (2) ☐ Poor (1) ☐
Lelei atoatoa *Lelei tele* *Lelei* *Lelei feololo* *Le lelei*

46. How would you rate the comfort of the waiting room? (*E fa'apefea ona e togiina le lelei o le potu o lo'o fa'atalitali ai ae le'i faia le talavai*)

Excellent (5) ☐ Very Good (4) ☐ Good (3) ☐ Fair (2) ☐ Poor (1) ☐
Lelei atoatoa *Lelei tele* *Lelei* *Lelei feololo* *Le lelei*

47. How would you rate the atmosphere of the waiting room? *(E fa'apefea ona e togiina le si'osi'omaga o le potu o lo'o fa'atali ai.*)

Excellent (5) ☐ Very Good (4) ☐ Good (3) ☐ Fair (2) ☐ Poor (1) ☐
Lelei atoatoa *Lelei tele* *Lelei* *Lelei feololo* *Le lelei*

48. How would you rate the location of the clinic? (*E fa'apefea ona e togiina le nofoaga o lo'o i ai le potu talavai?)*

Excellent (5) ☐ Very Good (4) ☐ Good (3) ☐ Fair (2) ☐ Poor (1) ☐
Lelei atoatoa *Lelei tele* *Lelei* *Lelei feololo* *Le lelei*

49. How would you rate the length of time you wait to see your doctor when you have an appointment? (*E fa'apefea ona e togiina le umi o le taimi o lo'o e fa'atali ai e vaai ai le foma'i pe a fai sou talavai?)*

Excellent (5) ☐ Very Good (4) ☐ Good (3) ☐ Fair (2) ☐ Poor (1) ☐
Lelei atoatoa *Lelei tele* *Lelei* *Lelei feololo* *Le lelei*

50. How would you rate the waiting time to get an appointment (between the time you call and come in)? *(E fa'apefea ona e togiina le taimi o lo'o e fa'atali e fai ai sou talavai fa'atulagaina (E aofia ai le taimi na e valaau mai ai ma le taimi na e sūsū mai ai totonu*)

Excellent (5) ☐	Very Good (4) ☐	Good (3) ☐	Fair (2) ☐	Poor (1) ☐
Lelei atoatoa	*Lelei tele*	*Lelei*	*Lelei feololo*	*Le lelei*

51. How would you rate the clinic when thinking about the hours it is open? (*E fa'apefea ona e togiina ia itula o lo'o matala ai le potu talavai*?)

Excellent (5) ☐	Very Good (4) ☐	Good (3) ☐	Fair (2) ☐	Poor (1) ☐
Lelei atoatoa	*Lelei tele*	*Lelei*	*Lelei feololo*	*Le lelei*

52. How would you rate the availability of nutritional services (people who can talk to you about what to eat during pregnancy)? (*E fa'apefea ona e togiina le i ai o tagata o lo'o mafai ona talanoa atu ia te oe auā lou taumafai i le taimi o le ma'itaga*.)

Excellent (5) ☐	Very Good (4) ☐	Good (3) ☐	Fair (2) ☐	Poor (1) ☐
Lelei atoatoa	*Lelei tele*	*Lelei*	*Lelei feololo*	*Le lelei*

53. How would you rate the availability of doctors? (*E fa'apefea ona e togiina le taimi avanoa o foma'i?)*

Excellent (5) ☐	Very Good (4) ☐	Good (3) ☐	Fair (2) ☐	Poor (1) ☐
Lelei atoatoa	*Lelei tele*	*Lelei*	*Lelei feololo*	*Le lelei*

54. How would you rate the helpfulness of advice you have received from the prenatal clinic during your pregnancy?(*E fa'apefea ona e togiina le aogā o fautuaga na e mauaina mai le potu talavai o tinā ma'itaga mo lenei ma'itaga*?)

Excellent (5) ☐	Very Good (4) ☐	Good (3) ☐	Fair (2) ☐	Poor (1) ☐
Lelei atoatoa	*Lelei tele*	*Lelei*	*Lelei feololo*	*Le lelei*

55. On average, how long do you spend at the prenatal care clinic for each appointment? (O le a umi e te nofo ai i le potu talavai o tinā ma'itaga mo ou talavai ta'itasi?)

0-30 minutes(*minute*) (0) ☐ 30 minutes(*minute*) – 1 hour(*itula*) (1) ☐

1 hour (*itula*) – 1 hour(*itula*) 30 minutes(*minute*) (2) ☐

1 hour(*itula*) 30 minutes(*minute*) – 2 hours(*itula)* (3) ☐ More than 2 hours (*E silia ma le 2 itula*) (4) ☐

56. On average, how many minutes after you arrive at the clinic do you check in / see the nurse?(*E fia minute talu ona e o'o atu i le potu talavai e te alu ai e vaai le 'au tausi ma'i?)*

_______________ minutes (*minute*)

57. On average, how many minutes after you arrive at the clinic do you see the doctor?(*E fia minute talu ona e o'o atu i le potu talavai, e te alu ai e vaai le foma'i*?)

_______________ minutes(*minute*)

58. On average, how many minutes do you spend talking with the doctor / being examined?(*E fia minute e te talanoa ai ma le foma'i o lo'o faia ou su'ega*?)

______________ minutes (*minute*)

59. If there were educational sessions at the prenatal clinic about health during pregnancy, where nurses would talk with <u>you and a group of other women</u> about pregnancy health and caring for your baby while you waited to see the doctor for your regular appointment, would you be interested in attending? (*E te fia auai pe afai o le a i ai ni porokalama i le potu talavai o tinā ma'itaga o le a talanoaina ai e le 'au tausi ma'i le tausiga o lou pepe a le'i faia se talavai ma le foma'i*?)

No(*Leai*) (0) ☐ Yes (1)(*Ioe*) ☐ Maybe (*Pei uma*) (2) ☐

If you would like to provide any other information or comment on your prenatal care experience please do so here (*A i ai nisi ou fa'amatalaga e fa'atatau i lou tausiga i le potu talavai o tinā ma'itaga, fa'amolemole tusi ifo i laina o i lalo ifo*)

____

____

____

____

____

____

You have completed this questionnaire. Please hand the questionnaire back to one of the study staff. Thank you very much for your participation!

(Ua ma'ea ona e fa'atumuina lenei pepa fesili. Fa'amolemole tauaao atu lenei pepa fesili i se tasi o le 'au su'esu'e. Fa'afetai tele lava mo lou auai!)
